# PRMT5 promotes cell proliferation by inhibiting BTG2 expression via the ERK signaling pathway in hepatocellular carcinoma

**DOI:** 10.1002/cam4.1360

**Published:** 2018-02-14

**Authors:** Hai Jiang, Yue Zhu, Zhenyu Zhou, Junyang Xu, Shaowen Jin, Kang Xu, Heyun Zhang, Qing Sun, Jie Wang, Junyao Xu

**Affiliations:** ^1^ Guangdong Province Key Laboratory of Malignant Tumor Epigenetics and Gene Regulation Research Center of Medicine Sun Yat‐Sen Memorial Hospital Sun Yat‐Sen University Guangzhou 510120 China; ^2^ Department of Hepatobiliary Surgery Sun Yat‐Sen Memorial Hospital Sun Yat‐Sen University Guangzhou 510120 China; ^3^ Department of Neurology Forth Affiliated Hospital Guangzhou Medical University Guangzhou 510000 China; ^4^ Department of Pathology Sun Yat‐Sen Memorial Hospital Sun Yat‐Sen University Guangzhou 510120 China; ^5^ Department of Vascular and Thyroid Surgery Sun Yat‐Sen Memorial Hospital Sun Yat‐Sen University Guangzhou 510120 China

**Keywords:** BTG2, cell cycle, hepatocellular carcinoma, PRMT5, Prognostic marker

## Abstract

Increasing evidence suggests that PRMT5, a protein arginine methyltransferase, has roles in cell growth regulation and cancer development. However, the role of PRMT5 in hepatocellular carcinoma (HCC) progression remains unclear. Here, we showed that PRMT5 expression was frequently upregulated in HCC tissues, and its expression was inversely correlated with overall survival in HCC patients. PRMT5 knockdown markedly inhibited in vitro HCC proliferation and in vivo tumorigenesis. We revealed that the mechanism of PRMT5‐induced proliferation was partially mediated by BTG downregulation, leading to cell cycle arrest during the G1 phase in HCC cells. Ectopic BTG2 overexpression decreased HCC growth, caused cell cycle arrest at the G1 phase, and downregulated Cyclin D1 and Cyclin E1 protein expression. Furthermore, we found that PRMT5‐induced ERK phosphorylation regulated BTG2 expression in HCC cells, whereas pretreatment with a selective ERK1/2 inhibitor (PD184352) significantly reversed the effect of PRMT5 on BTG2 expression. Our results indicated that PRMT5 promotes HCC proliferation by downregulating BTG2 expression via the ERK pathway.

## Introduction

Hepatocellular carcinoma (HCC) is the third leading cause of cancer‐related mortality worldwide, causing approximately 600,000 deaths each year 1. In China, HCC is the third leading cause of cancer death and the fourth most frequently diagnosed cancer 2. Radical surgical resection of tumors and liver transplantation are the curative treatments for liver cancer. However, because of its late symptom presentation, most HCC patients are not candidates for surgical resection or liver transplantation, and the therapeutic options for these advanced HCC patients are limited. Currently, sorafenib is the only FDA‐approved drug for advanced liver cancer. However, the clinical benefits of sorafenib remain unacceptable 3, and new targets for HCC therapy are urgently needed.

Protein arginine methyltransferase 5 (PRMT5) is the major type II arginine methyltransferase and catalyzes the symmetric methylation of histone and nonhistone proteins. Recently, accumulating evidence has shown that PRMT5 modulates cell growth and transformation. Through its methyltransferase ability, PRMT5 regulates the expression of a wide spectrum of target genes 4. PRMT5 methylates the histones H3R8 and H4R3, leading to cell cycle gene silencing (CDKN2A and CCNE) and tumor suppressor gene silencing (ST7, NM23, RBL2, and SMAD7) 4, 5, 6. Additionally, PRMT5 methylates nonhistone proteins. For example, an experiment using MCF‐7 breast cancer cells showed that inhibiting PRMT5 decreased p53 stability and the expression of p53 target genes 7. PRMT5 knockdown inhibits proliferation and cell cycle transition from the G1 to S phase, and a large number of corroborating studies have shown that PRMT5 levels are elevated in various cancers, including gastric, colorectal, lung, lymphoma, leukemia, and liver cancers 5, 8, 9, 10, 11. However, the role of PRMT5 in HCC progression has not been fully elucidated. Identifying its downstream targets will aid in elucidating the mechanism of PRMT5 in liver cancer development.

B‐cell translocation gene 2 (BTG2) was first identified in 1991 in rat PC12 cells induced by nerve growth factor and in mouse 3T3 fibroblasts stimulated with tetradecanoylphorbol acetate 12, 13. BTG2 belongs to a family of antiproliferation genes 14. BTG2 overexpression is associated with cell cycle arrest from G1 to S phase by suppressing Cyclin B1, Cyclin D1, and Cyclin E1 15, 16. Numerous studies have shown that as a tumor suppressor, BTG2 is inhibited in human cancers, such as skin, lung, breast, and gastric cancers, and in bladder carcinoma 17, 18, 19, 20. To date, mutations in BTG2 have not been detected, indicating that epigenetic modification might be the main mechanism by which BTG2 expression is modulated. Recently, the chromatin‐modifying enzyme SETD1A was found to regulate BTG2 expression through an miRNA network. However, whether arginine methyltransferases regulate BTG2 expression in HCC is unknown.

In this study, we found that PRMT5 was overexpressed in HCC and that PRMT5 promoted in vitro and in vivo cancer cell growth. We detected an inverse correlation between PRMT5 and BTG2 expressions. Inhibition of PRMT5 elevated BTG2 expression by activating ERK signaling. Moreover, silencing BTG2 expression reversed the effects induced by PRMT5 knockdown in HCC cells.

## Materials and Methods

### Cell lines and reagents

HEK 293T cells and the HCC cell lines LO2, Huh7, Bel7402, PLC/PRF/5, HepG2, MHCC97H, and SK‐Hep1 were purchased from the Chinese Academy of Sciences Cell Bank of Type Culture Collection (CBTCCCAS). The cell lines were maintained in DMEM (Gibco, Carlsbad, CA) supplemented with 10% fetal bovine serum (Gibco) in an atmosphere of 5% CO_2_ at 37°C. ERK1/2 inhibitor (PD184352) and the selective PRMT5 inhibitor GSK591/EPZ015866 were purchased from Selleck (Houston, TX, USA).

### Patient samples and tissue microarray

Hepatocellular carcinoma and corresponding adjacent normal liver tissue samples were collected after HCC tumor resection at Sun Yat‐sen Memorial Hospital, Guangzhou. In total, 138 HCC patients who received curative resection between 2005 and 2012 at Sun Yat‐sen Memorial Hospital were enrolled in this study. The study protocol was approved by the Institute Review Board of the University of Sun Yat‐sen, and written informed consent was obtained from each patient. HCC patients with a distinctive pathologic diagnosis, no preoperative systemic or local treatment, and complete follow‐up data were selected for inclusion in this study. The pTNM classification for HCC was defined according to The American Joint Committee on Cancer/International Union Against Cancer staging system (7th edition, 2010). To detect PRMT5 mRNA levels, 48 pairs of HCC samples and corresponding adjacent normal liver tissues were collected after surgical resection and stored in liquid nitrogen. A tissue microarray (TMA) consisting of 138 pairs of HCC tissues and corresponding adjacent normal liver tissues was constructed by Shanghai Biochip Company (Shanghai, China).

### cDNA preparation and RT‐qPCR

Total RNA was extracted using TRIzol reagent (Takara, Dalian, China) according to the manufacturer's instructions. Reverse transcription was performed using **PrimeScript RTase** (Takara) according to the manufacturer's protocol. The expression levels of PRMT5 and BTG2 mRNA were determined with RT‐qPCR using Premix Ex Taq (Takara) according to the manufacturer's instructions and normalized to the expression levels of the endogenous control, GAPDH. The primers used for RT‐qPCR were as follows:

GAPDH: sense 5′‐GAAGGTGAAGGTCGGAGTCAACG‐3′ and antisense 5′‐TGCCATGGGTGGAATCATATTGG‐3′; PRMT5: sense 5′‐CCTGTGGAGGTGAACACAGT‐3′ and antisense 5′‐AGAGGATGGGAAACCATGAG‐3′; BTG2: sense 5′‐ATGAGCCACGGGAAGGGAA‐3′ and antisense 5′‐TTGGACGGCTTTTCGGGAA‐3′.

### Immunohistochemistry

The expression of PRMT5 and BTG2 proteins was assessed with immunohistochemistry in a tissue microarray using rabbit polyclonal antibodies specifically against PRMT5 (Abcam, Cambridge, USA ab109451), BTG2 (Abcam, Cambridge, USA ab85051), and Ki67 (Abcam, Cambridge, USA ab15580). Immunohistochemistry staining was performed using the Dako Envision Plus System (Dako, Carpinteria, CA) according to the manufacturer's protocol.

Each sample was independently scored by two pathologists who were blinded to all the patient clinical data. The intensity of staining was scored as 0 (negative), 1 (weak), 2 (moderate), or 3 (strong). Based on the percentage of positive tumor cells, the extent of staining was scored 0 (negative), 1 (1–25%), 2 (26–50%), 3 (51–75%), or 4 (76–100%). The final score for each slide was assessed by multiplying the scores for intensity and extent of staining. The slide was considered negative if the score was <5 and positive if the score was above 5.

### Establishment of cell lines stably knocking down PRMT5 and overexpressing BTG2

PRMT5 (human) shRNA lentiviral vectors were purchased from Shanghai Genechem Co. Ltd. (Shanghai, China), and BTG2 (human) overexpression lentiviral vectors were purchased from Shanghai Generay Biotech Co. Ltd. Lentivirus was produced by cotransfection of 293T cells with viral vectors (pCMV‐MDL, pCMV‐REV, and pCMV‐VSVG). Medium containing virus was collected and concentrated using PEG8000 (Sigma‐Aldrich). Infected cells were treated with puromycin (final concentration: 1 *μ*g/mL) for 2 weeks of selection.

### MTT proliferation assay

Cell proliferation was analyzed using an MTT assay according to the manufacturer's protocol. In brief, 1000 cells in 100 *μ*L/well were seeded in triplicate in 96‐well plates for 1–72 h at 37°C in 5% CO_2_. Then, 20 *μ*L of MTT solution was added to each well and incubated with the cells for 4 h. Then, 150 *μ*L DMSO was added to each well and incubated for 15 min. Absorbance at 570 nm was measured with a microplate reader.

### Colony formation assay

Hepatocellular carcinoma cells were seeded at 1000 cells per well into six‐well plates. The number of colonies was assessed using crystal violet staining after 2 weeks. Three independent experiments were performed, and the results are expressed as the mean ± SD.

### Cell cycle analysis

Post‐treatment, cells were harvested, washed twice with PBS, fixed in 75% EtOH, and stained with PI. Stained cells were analyzed with a flow cytometer using Beckman Coulter (Brea, CA) and **ModFit** (Verity, ME) software.

### Protein extraction and western blotting

Cells were harvested and lysed in RIPA buffer. Proteins from lysed cells were separated from 8 to 12% SDS‐PAGE gels and transferred to 0.22 *μ*m polyvinylidene difluoride membranes (Millipore, Frankfurt, Germany). Then, 5% milk in TBST was used to block nonspecific binding sites. The membranes were incubated with primary antibodies overnight at 4°C. Then, the blots were washed with 0.1% TBST three times and incubated with horseradish peroxidase‐conjugated antibodies. The protein was detected using SuperSignal West Dura (Thermo, Waltham, USA). The antibodies used for western blotting were against GAPDH (sc‐293335), PRMT5 (Abcam ab109451), BTG2 (Abcam ab85051), Cyclin D1 (Abcam ab134175), Cyclin E1 (sc‐377100), p44/42 MAPK (Erk1/2) (CST #4695), phospho‐p44/42 MAPK (Erk1/2) (Thr202/Tyr204) (CST #4370), phospho‐c‐Raf (Ser338) (CST #9427), Histone H4 symmetric dimethyl R3 (H4R3me2s) (Abcam ab5823), and Histone H3 (Abcam ab1791).

### Nuclear extracts

Nuclear extracts were obtained with an N‐PER kit (Pierce, Waltham, USA) according to the manufacturer's instructions.

### RNA interference

Small interfering RNA (siRNA) specifically targeting BTG2 and scramble siRNA (GenePharma, Shanghai, China) were transfected into cells using Lipofectamine 3000 (Invitrogen, USA) according to the manufacturer's instructions.

### Xenograft tumors in nude mice

A xenograft tumor growth assay was performed by subcutaneously injecting Huh7 cells (5 × 106) stably transduced with lentiviruses encoding scrambled shRNA (shControl) or shRNA targeting PRMT5 (shPRMT5) in 0.2 mL of phosphate‐buffered saline (PBS) into the right shoulder of 6‐week‐old BALB/c nude mice. All the mice were sacrificed 4 weeks after injection. Tumors were removed and weighed. All the animal experiments were performed according to the institutional standard guidelines at Sun Yat‐sen University. The IRB number is IACUC‐DD‐17‐0803, and the animal study approved number is SYXK (Yue) 2016‐0112.

### Statistical analysis

All the statistical analyses were performed with SPSS 19.0 or GraphPad Prism 6.0. Paired *t*‐tests were used to compare xenograft tumor size, and PRMT5 mRNA levels and immunostaining scores in paired clinical samples. Pearson's chi‐square test was used to analyze clinical correlations. Kaplan–Meier plots and log‐rank tests were used to assess overall survival. Univariate and multivariate survival analyses were performed using Cox proportional hazards regression. Statistical results were considered significant when *P *<* *0.05.

## Results

### PRMT5 overexpression in HCC tissues and correlation with patient clinicopathological features and survival

**Figure 1:**
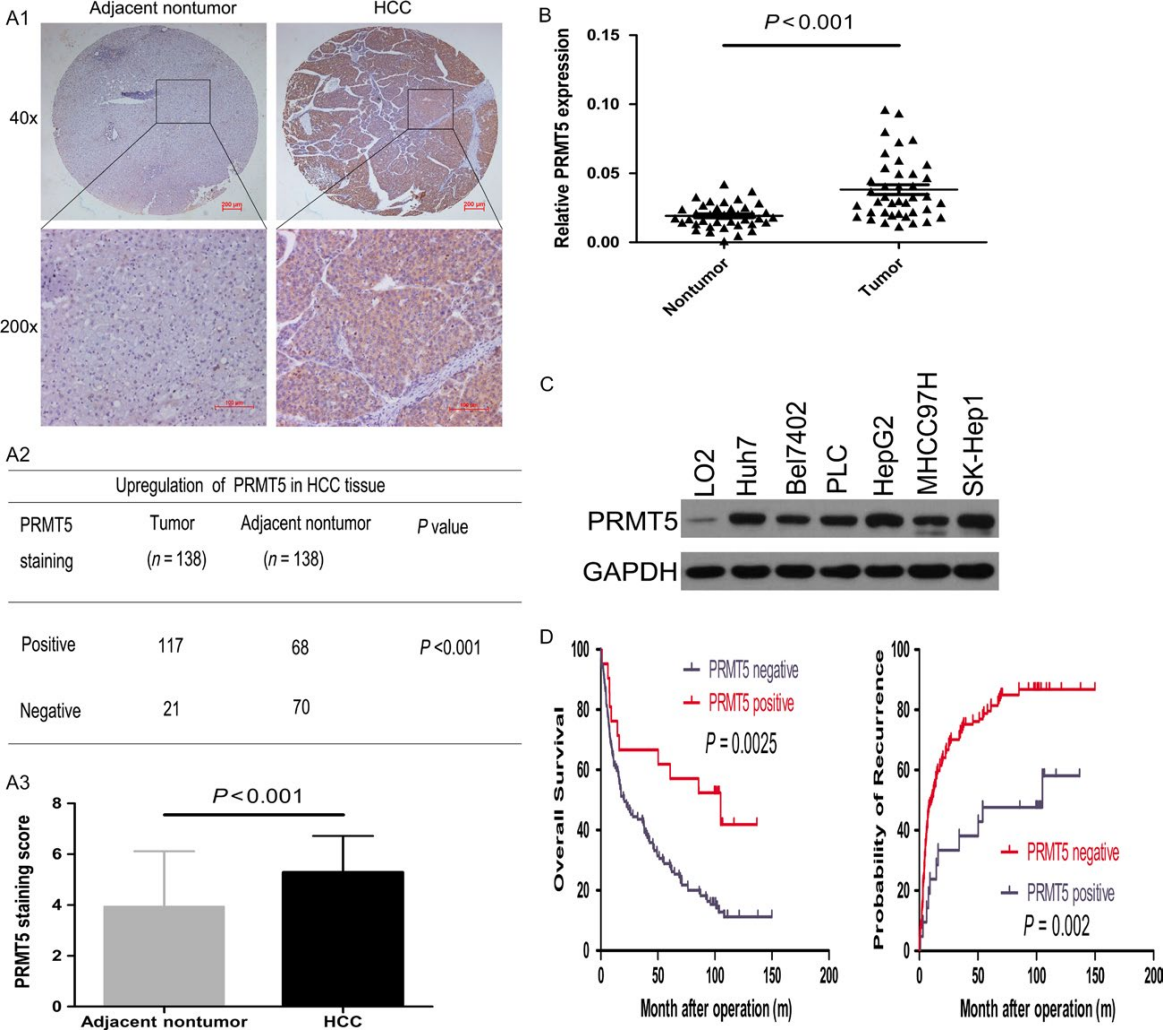
PRMT5 overexpression in HCC tissues and correlation with patient clinicopathological features and survival. (A1–A3) The IHC staining of PRMT5 in adjacent nontumorous tissues and HCC tissues and statistical analysis of staining scores were presented. (B) The mRNA levels of PRMT5 in paired HCC tissues were detected by real‐time PCR assay. (C) The protein levels of PRMT5 in HCC cell lines were detected by western blot assay. (D) Kaplan–Meier analysis of the correlation between PRMT5 expression and overall survival or recurrence rate in HCC patients (*n* = 138).

To investigate PRMT5 expression in HCC, we performed immunohistochemical staining on tumor tissue microarrays (TMAs) containing pairs of HCC specimens and corresponding pericancerous liver tissues from an HCC cohort (*n* = 138). Immunostaining for PRMT5 was observed predominantly in the cytoplasm but was occasionally seen in nuclei (Fig. 1A1). Of the 138 HCC cases, 117 cases (84.7%) were positive for PRMT5 expression with a score>5; in contrast, 68 of 138 cases (49.2%) had positive scores in adjacent liver tissues (Fig. 1A2). Taking the staining intensity into account, the PRMT5 staining score in HCC was also higher than that in adjacent nontumor tissues (Fig. 1A3). We confirmed PRMT5 upregulation in an additional 48 paired HCC tissues using real‐time PCR (Fig. 1B). Furthermore, PRMT5 protein levels were increased in HCC cell lines compared to those in a normal liver cell line (LO2) (Fig. 1C). Correlation analysis demonstrated that PRMT5 expression was positively correlated with tumor size (Table 1). Kaplan–Meier analysis showed that enhanced PRMT5 expression was correlated with reduced overall survival and higher recurrence rates for patients with HCC (Fig. 1D). Multivariate Cox regression analysis determined that PRMT5 overexpression is an independent and significant predictor of survival and recurrence in HCC after surgical resection (Table 2).

**Table 1:** Correlation between PRMT5 expression and patient's clinicopathologic features in HCCs. *P < 0.05

Clinicopathological variables	Case number (*n* = 138)	Tumor PRMT5 expression		*P* value
		Negative (*n* = 21)	Positive (*n* = 117)	
Age				
≤52 years	74	9	65	0.283
>52 years	64	12	52	
Sex				
Female	21	2	19	0.430
Male	117	19	98	
Serum AFP				
≤20 ng/mL	40	7	33	0.633
>20 ng/mL	98	14	84	
HBsAg				
Negative	118	20	98	0.299
Positive	20	1	19	
Cirrhosis				
Present	108	16	92	0.465
Absent	32	5	27	
Tumor size				
≤5 cm	53	15	43	0.003*
>5 cm	85	6	74	
Vascular invasion				
Present	56	7	49	0.463
Absent	82	14	68	
Tumor encapsulation				
Present	74	13	61	0.409
Absent	64	8	56	
Tumor differentiation				
I–II	101	15	86	0.214
III–IV	38	6	31	
TNM stage				
I–II	74	12	62	0.283
III–IV	64	9	54	

**Table 2:** Univariate and multivariate analysis of factors associated with survival and recurrence of 138 HCCs. *P < 0.05

Variables	Survival						Recurrence					
	Univariate analysis			Multivariate analysis			Univariate analysis			Multivariate analysis		
	HR	95% CI	*P* value	HR	95% CI	*P* value	HR	95% CI	*P* value	HR	95% CI	*P* value
Age (≤52 vs. >52)	1.173	0.815–1.689	0.390				1.250	0.869–1.797	0.230			
Sex (female vs. male)	0.827	0.486–1.407	0.484				0.838	0.493–1.425	0.515			
Serum AFP (≤20 vs. >20 ng/mL)	1.153	0.782–1.700	0.472				1.159	0.787–1.708	0.455			
HBsAg (negative vs. positive)	1.076	0.644–1.798	0.781				0.910	0.581–1.623	0.910			
Tumor encapsulation (present vs. absent)	0.762	0.530–1.094	0.141				0.705	0.491–1.012	0.058			
Cirrhosis (absent vs. present)	1.277	0.827–1.974	0.270				1.253	0.811–1.936	0.310			
Tumor size (≤5 vs. >5 cm)	1.690	1.164–2.453	0.006*				1.658	1.144–2.403	0.008*			
Vascular invasion (absent vs. present)	1.660	1.150–2.396	0.007*				1.587	1.101–2.289	0.013*			
Tumor differentiation (I‐II vs. III‐IV)	0.909	0.598–1.382	0.656				1.069	0.705–1.620	0.753			
TNM stage (I‐II vs. III‐IV)	1.877	1.304–2.700	0.001*	1.959	1.359–2.825	0.000*	1.783	1.240–2.565	0.002*	1.797	1.250–2.585	0.002*
Tumor PRMT5 expression (negative vs. positive)	1.757	1.036–2.981	0.037*	1.893	1.113–3.219	0.019*	1.786	1.052–3.031	0.032*	1.810	1.066–3.072	0.028*

### PRMT5 knockdown inhibits in vitro and in vivo HCC cell proliferation

**Figure 2:**
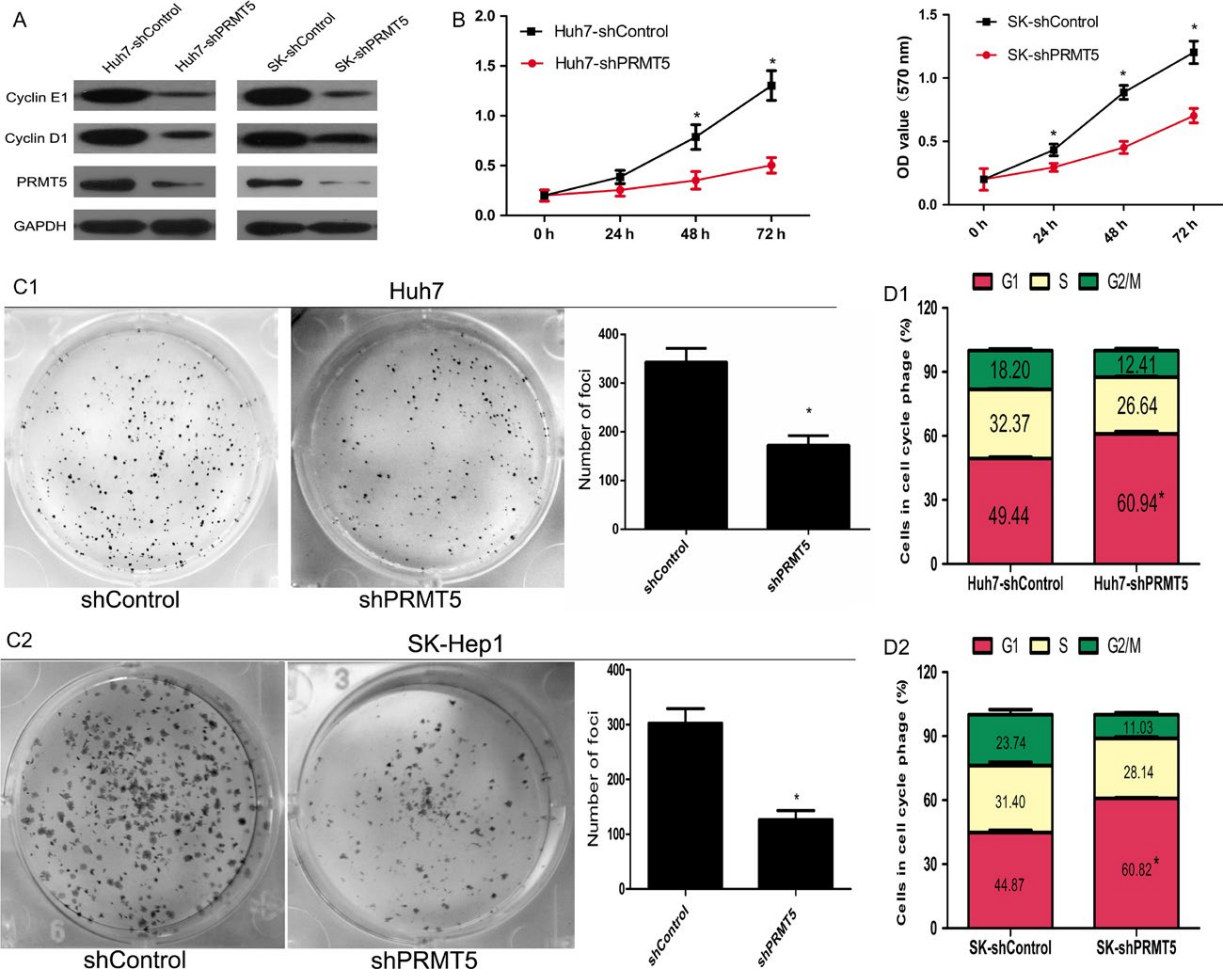
PRMT5 knockdown inhibits in vitro and in vivo HCC cell proliferation. (A) Expression level of PRMT5 protein in HCC cells stably expressed shRNA sequence against PRMT5 (shPRMT5) and nontarget control (shControl). (B) Knockdown of PRMT5 inhibited HCC cell proliferation, as detected by MTT assay. (C) Decreased foci formation in monolayer culture induced by PRMT5 inhibition. Right panel shows the quantitative analyses of foci numbers. (D) Knockdown of PRMT5 in HCC cells increased the G1 fraction, as detected by flow cytometry. (All the experiments were repeated three times and the results are presented as mean ± standard deviation, *P < 0.05 indicates significant difference in independent Student's *t*‐test).

To explore the role of PRMT5 in HCC cell proliferation, we established two cell lines, Huh7‐shPRMT5 and SK‐shPRMT5, which were stably transduced with shPRMT5 lentivirus. Significant inhibition of endogenous PRMT5 expression in Huh7 and SK‐Hep1 cells was confirmed by western blotting analysis (Fig. 2A). MTT (Fig. 2B) and colony formation (Fig. 2C) assays demonstrated that stable silencing of PRMT5 significantly decreased HCC cell proliferation. Flow cytometry analysis showed that PRMT5 inhibition induced cell cycle arrest at the G1 phase in HCC cells (Fig. 2D).

**Figure 3:**
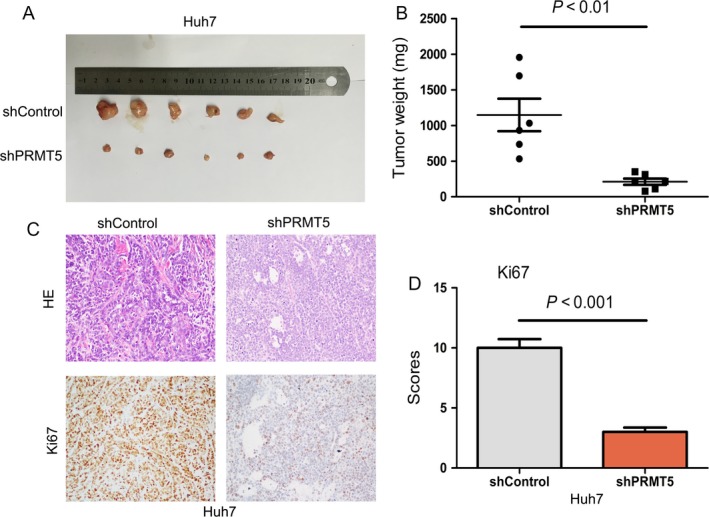
Inhibition of PRMT5 suppresses HCC growth in vivo. (A) Four weeks after HCC cells transplantation, tumors were harvested and photographed. (B) All harvested tumors were weighted in both groups. (C) Representative images of IHC staining of Ki67 showed that PRMT5 inhibition decreased tumor proliferation in xenografted tumors. (D) Quantification of IHC score for Ki67 staining was analyzed by Student's *t*‐test.

To determine whether PRMT5 provided an in vivo growth advantage to HCC cells, xenograft studies were performed. Tumors in mice injected with cells stably knocking down PRMT5 were smaller and lighter than those in the control group (Fig. 3A and B). In addition, xenograft tumor sections were stained for Ki67 to observe tumor proliferation status, which indicated that tumors derived from cells with stably silenced PRMT5 showed significantly reduced proliferation (Fig. 3C and D). Together, these results showed that inhibition of PRMT5 reduced cancer cell proliferation and tumor growth.

### PRMT5 downregulates BTG2 expression in HCC cells

**Figure 4:**
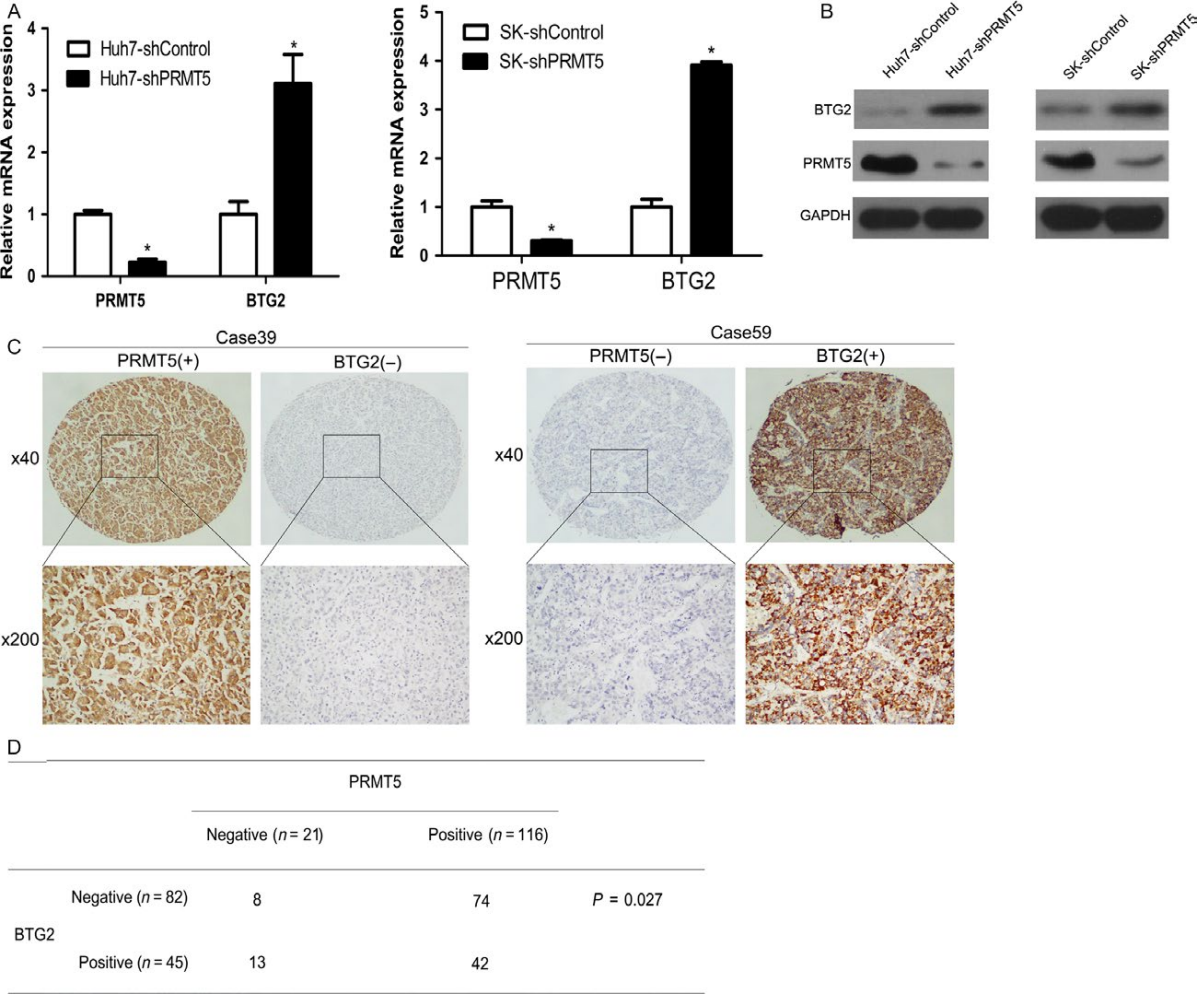
Knockdown of PRMT5 enhances BTG2 expression in Huh7 and SK‐Hep1 cells. (A) Real‐time PCR and (B) western blot showed upregulated expression of BTG2 in Huh7‐shPRMT5 and SK‐shPRMT5 cells. (C and D) IHC analysis showed inverse correlation of PRMT5 expression and BTG2 expression in consecutive HCC tissue sections.

By analyzing the GEO database, we found that BTG2 levels were significantly upregulated in several cancers, including lung cancer (www.ncbi.nlm.nih.gov/geo/query/acc.cgi?acc=GSE56757) and prostate cancer (www.ncbi.nlm.nih.gov/geo/query/acc.cgi?acc=GSE65965), after knockdown of PRMT5. However, the regulation of BTG2 by PRMT5 in HCC has not been verified. Therefore, we performed RT‐qPCR and western blotting in HCC cells after PRMT5 knockdown. Specific knockdown of PRMT5 significantly increased BTG2 mRNA levels in Huh7‐shPRMT5 cells and SK‐shPRMT5 cells by three‐ to fourfold when compared with those in the scrambled shRNA‐treated group (Fig. 4A). Likewise, BTG2 protein levels were significantly upregulated after PRMT5 inhibition in HCC cells (Fig. 4B). Furthermore, immunohistochemistry showed that PRMT5 expression was negatively correlated with BTG2 expression in HCC tissues (Fig. 4C and D).

### PRMT5 downregulates BTG2 expression through ERK signaling

**Figure 5:**
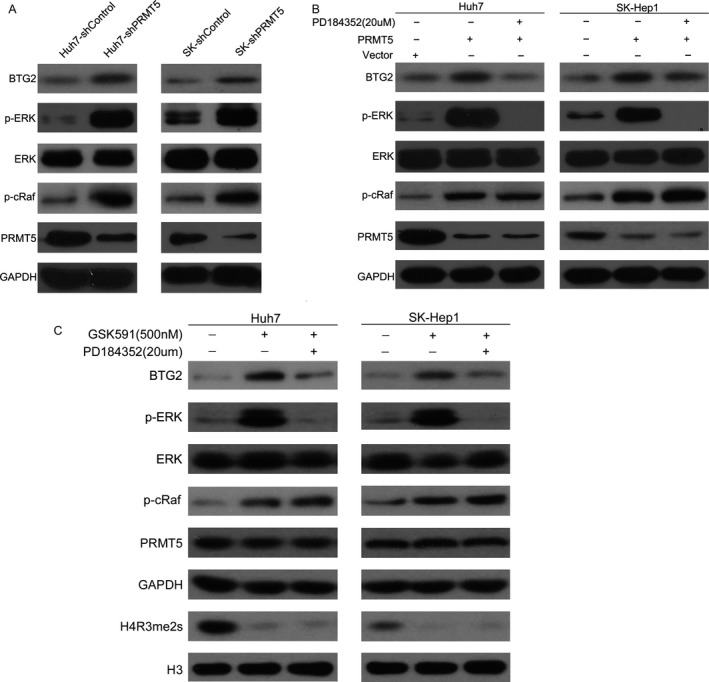
Inhibition of PRMT5 or its activity upregulates BTG2 expression through the phosphorylation of ERK. (A and B) Western blot showed that knockdown of PRMT5 increased the phosphorylation of Raf and ERK and protein level of BTG2 in HCC cells, while inhibition of ERK phosphorylation by 24 h pretreatment with 20 *μ*M p‐ERK inhibitor PD184352 in shPRMT5 cells led to downregulation of BTG2 and p‐ERK. (C) Treatment of HCC cells with 500 nmol/L GSK591 for 4 days led to significant loss of PRMT5‐catalyzed methylarginine on H4 (H4R3me2s), the upregulation of BTG2, and the phosphorylation of ERK. Inhibition of ERK phosphorylation by PD184352 treatment reversed these effects. H4R3me2s was used to detect the PRMT5 activity. Histone 3 was used as nuclear loading control

BTG2 has been reported to be regulated by ERK signaling in oral squamous cancer cells. Thus, we examined this possibility in HCC cancer cells. The levels of BTG2,p‐cRaf, and p‐ERK were both increased after PRMT5 knockdown in HCC cells (Fig. 5A). After HCC cells were pretreated with an ERK phosphorylation inhibitor (PD184352) for 24 h to block ERK activation, the increased BTG2 protein levels induced by PRMT5 inhibition were significantly suppressed (Fig. 5B). A previous study indicated that PRMT5 activity was necessary for its function, prompting us to treat HCC cells with a potent and specific PRMT5 chemical probe, GSK591. After treatment with 500 nmol/L GSK591 for 4 days, a significant loss of PRMT5‐catalyzed methylarginine on H4 (H4R3me2s), upregulation of BTG2, and phosphorylation of ERK were observed, whereas inhibiting ERK phosphorylation with PD184352 treatment reversed these effects (Fig. 5C). These data indicated that PRMT5 downregulates BTG2 expression through ERK signaling and demonstrates the requirement for PRMT5 activity in its HCC‐promoting function.

### BTG2 overexpression causes cell cycle arrest in the G1 phase and inhibits in vitro HCC proliferation

**Figure 6:**
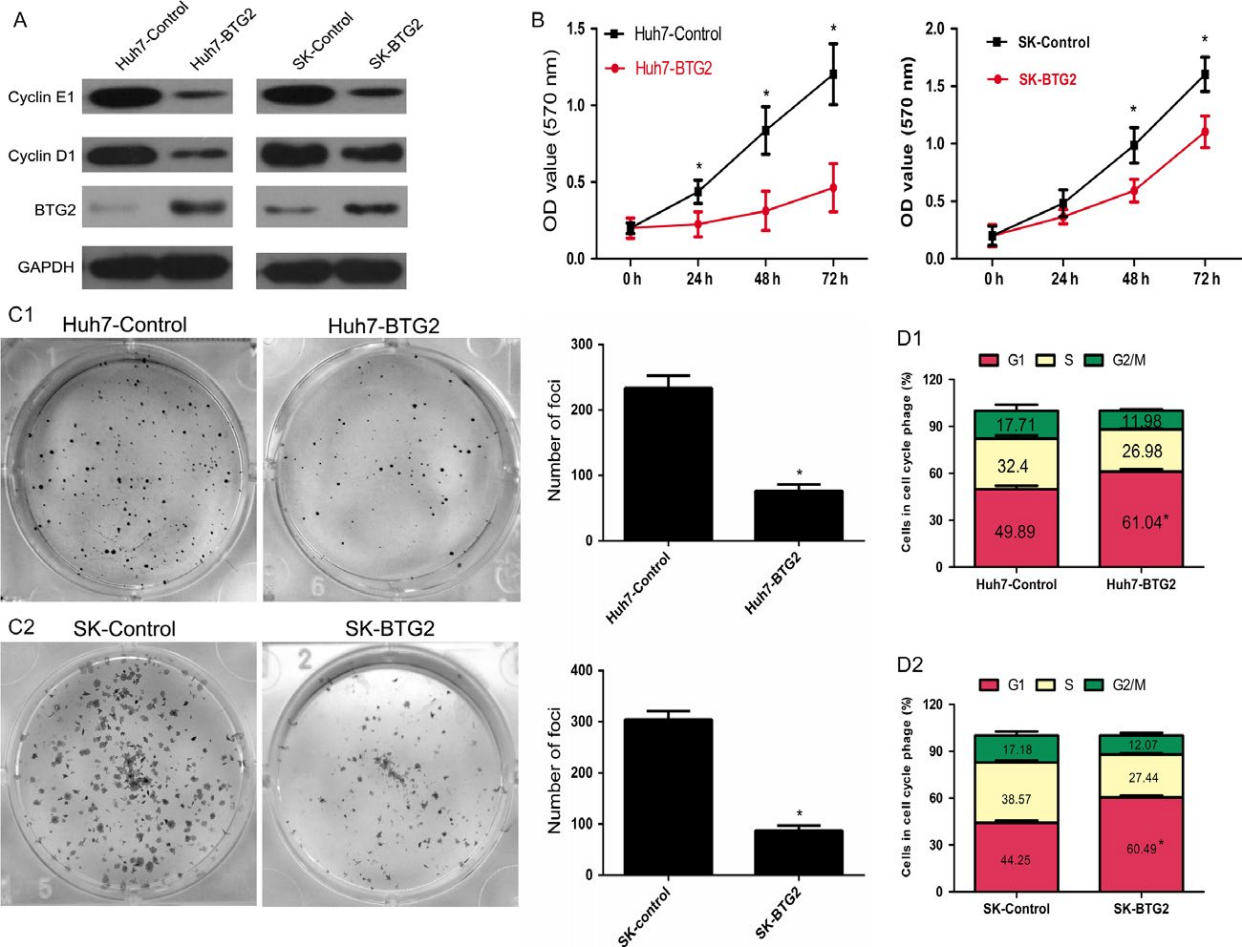
BTG2 overexpression causes cell cycle arrest in the G1 phase and inhibits in vitro HCC proliferation. (A) After stable transfection of BTG2, protein expression of BTG2 and Cyclin D1 in HCC cells were detected by western blot. (B) Cell growth was inhibited after overexpression of BTG2, as detected by MTT assay. (C) Decreased foci formation in monolayer culture induced by BTG2 overexpression. Right panel shows the quantitative analyses of foci numbers. (D) Enhanced BTG2 expression increased the G1 fraction, as detected by flow cytometry. (All the experiments were repeated three times, and the results are presented as mean ± standard deviation, *P < 0.05 indicates significant difference in independent Student's *t*‐test).

To investigate the role of BTG2 in HCC cells, we established two stable cell lines (Huh7‐BTG2 and SK‐BTG2) infected with BTG2‐overexpressing lentivirus (LV‐BTG2). BTG2 expression was confirmed by western blotting. BTG2 overexpression inhibited Cyclin D1 and Cyclin E1 expressions, as detected by immunoblotting analyses (Fig. 6A). The growth of Huh7‐BTG2 and SK‐BTG2 cells was significantly decreased compared to Huh7‐Control and SK‐Control cells as determined with an MTT assay (Fig. 6B). To assess the effect of BTG2 on tumorigenicity, a colony formation experiment was performed. Colony formation by Huh7‐BTG2 and SK‐BTG2 cells was significantly inhibited (Fig. 6C). Additionally, we analyzed changes in cell cycle distribution. In BTG2‐overexpressing HCC cells (Huh7‐BTG2 and SK‐BTG2 cells), the percentage of cells in the G1 phase was higher than that in the Huh7‐Control and SK‐Control cells. The quantitative analysis showed that Huh7‐BTG2 and SK‐BTG2 cells exhibited 11.1% and 16.2% increases in the G1 phase population. In contrast, the cell percentage in S and G2/M phases decreased in BTG2‐overexpressed cells (Fig. 6D1 and D2).

### PRMT5 promotes HCC proliferation in part by inhibiting BTG2 expression

**Figure 7:**
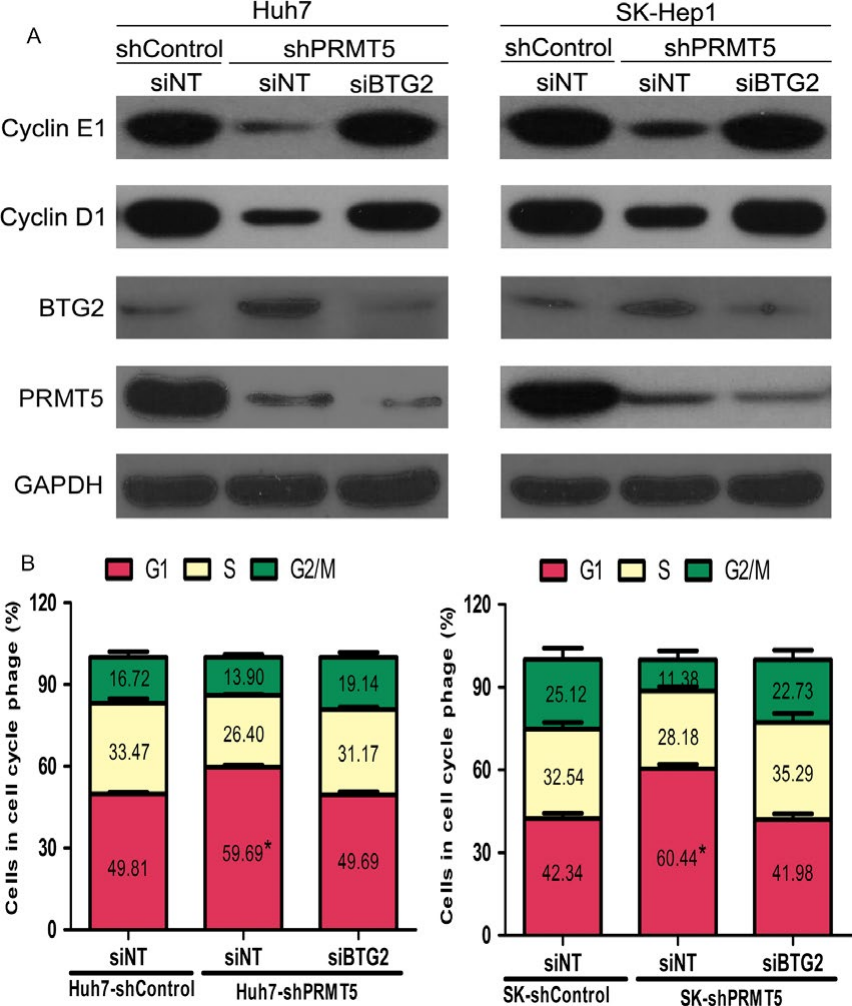
PRMT5 promotes HCC proliferation in part by inhibiting BTG2 expression. (A) shPRMT5‐infected HCC cells were transiently transfected with siRNAs (siNT) or siBTG2, and shControl‐infected cells transfected with siNT served as control. Western blot showed that knockdown of PRMT5 increased BTG2 expression and downregulated its downstream gene Cyclin D1 and Cyclin E1 levels, while inhibition of BTG2 with siBTG2 in shPRMT5 cells reversed this effect. (B) Cell cycle analysis of shPRMT5‐infected cells transfected with siRNA (siNT) or siBTG2 showed that BTG2 inhibition reversed the effect of cell cycle arrest at G1 phase in shPRMT5 HCC cells.

Considering that inhibition of PRMT5 induced BTG2 expression in HCC cells and that BTG2 inhibited proliferation in HCC cells, we speculated that knockdown of PRMT5 inhibited HCC cell growth by stimulating BTG2 expression. To verify this hypothesis, Huh7‐shPRMT5 cells and SK‐shPRMT5 cells were transfected with siRNA (siBTG2), and Huh7‐shControl cells and SK‐shControl cells were transfected with scrambled RNA (siNT). The protein levels of PRMT5, BTG2, Cyclin D1, and Cyclin E1 were detected by western blotting (Fig. 7A). PRMT5 inhibition significantly enhanced BTG2 expression, decreased Cyclin D1 protein levels, and arrested the cell cycle at the G1 phase. Depletion of BTG2 by siRNAs partially reversed this effect (Fig. 7B).

## Discussion

Accumulating evidence has demonstrated that HCC is a disease involving the accumulation of multiple epigenetic and genetic alterations 21. These alterations lead to tumor suppressor inactivation and the activation of oncogenes 4. PRMT5 belongs to the PRMT family of enzymes; these enzymes have been implicated in gene transcription, signal transduction, and DNA repair. PRMT5 is the major symmetric arginine methyltransferase and is ubiquitously expressed and regulates important cellular functions, including cell growth, proliferation, and differentiation 5, 22, 23, 24. Dysregulation of PRMT5 has been implicated in carcinogenesis, including lung cancer, gastric cancer, and prostate cancer 25, 26, 27.

In this study, we explored PRMT5 oncogenicity in HCC. We found that PRMT5 was frequently upregulated in HCC tissues and HCC cell lines compared with corresponding adjacent noncancerous tissues and a normal liver cell line (LO2), respectively. PRMT5 overexpression was associated with tumor size (*P *=* *0.003). In addition, patients positive for PRMT5 expression exhibited shorter overall survival time and recurrence time than patients negative for PRMT5 expression. Furthermore, multivariate analysis revealed that PRMT5 expression was an independent and significant risk factor for survival and recurrence following curative resection. These data suggested that PRMT5 might be an oncogene and a useful prognostic biomarker.

One of the procancer mechanisms of PRMT5 is the silencing of tumor suppressor genes. Previous studies have reported that PRMT5 regulates cell proliferation by silencing the tumor suppressor genes ST7, NM23, RBL2, and SMAD7 23, 28. However, the mechanism by which PRMT5 regulates cancer cell proliferation remains undetermined. By analyzing the GEO database, we found that the level of BTG2 was significantly upregulated in several cancers, including lung cancer and prostate cancer, after PRMT5 knockdown. Notably, BTG2 has been implicated in the growth of gastric cancer and liver cancer cells 15, 19. Importantly, our study demonstrated that BTG2 was significantly upregulated in PRMT5‐silenced HCC cells. In addition, immunohistochemistry showed that PRMT5 expression was inversely correlated with BTG2 protein expression in HCC tissues. These data demonstrate that BTG2 might be a PRMT5 target.

The MAPK pathway plays a crucial role in cell proliferation, differentiation, survival, and apoptosis 29, 30, 31, 32. A large number of reports have shown that ERK activation suppresses cell proliferation or causes cell death 33, 34. The amplitude and duration of the ERK signal play a key role determining the final biological outcome 34. Some reports have suggested that a large and sustained increase in ERK activation suppresses cell proliferation, whereas rapid and transient activation of ERK induces cell growth 35, 36. Pedro et al. 37 reported that inhibition of PRMT5 activity or mutation of RAF affected ERK signal amplitude and duration, redirecting PC12 cells from proliferation to differentiation. Recently, Lee et al. 16 reported that epigallocatechin‐3‐gallate increased BTG2 levels via p38 and the ERK1/2 signaling pathways, which suppressed OSCC cell growth. Therefore, we hypothesized that PRMT5 regulated BTG2 expression via the ERK pathway. Our results showed that PRMT5 inhibition in HCC cells significantly induced p‐Raf, p‐ERK1/2, and BTG2 expressions. The BTG2 protein increase induced by PRMT5 inhibition was blocked when cells were pretreated with an ERK inhibitor (PD0325901). In addition, Chen et al. 27 reported that PRMT5 activity was necessary for its phenotypes. To provide additional evidence for this hypothesis, we used GSK591 to block PRMT5 activity. Our data indicated that blocking PRMT5 activity had the same effects as inhibiting PRMT5 expression. Collectively, our data showed that downregulation of PRMT5 or its activity enhanced BTG2 expression via the ERK1/2 MAPK signaling pathway.

Consistent with a previous report, our study showed that BTG2 upregulation significantly induced cell cycle arrest in the G1 phase and suppressed proliferation in HCC cells 15. Because PRMT5 inhibition decreased HCC proliferation and induced BTG2 expression, we hypothesized that BTG2 functions downstream of PRMT5 in the proliferative regulation of HCC cells. Our further cell cycle and western blotting assays verified that PRMT5 inhibition induced G1/S arrest in HCC cells by inhibiting Cyclin D1 and Cyclin E1, whereas pretreatment of cells with siRNA targeting BTG2 in shPRMT5‐HCC cells reversed this effect. These results indicated that the regulatory effect of PRMT5 on the cell cycle and proliferation in HCC was partially mediated by BTG2.

In conclusion, we found that PRMT5 was frequently overexpressed in HCC and that its enhanced expression was associated with a poor prognosis. PRMT5 inhibition induced cell cycle arrest in the G1 phase and inhibited in vitro and in vivo proliferation. PRMT5 inhibition increased BTG2 expression through ERK phosphorylation, thus inducing cell cycle arrest and suppressing proliferation in HCC cells. These results suggest that PRMT5 might be a potential new therapeutic target in HCC.

## Conflicts of Interest

The authors declare that they have no conflicts of interest.
